# Anti-Glomerular Basement Membrane Disease in a 10-year-old Child: A Case Report

**DOI:** 10.31729/jnma.8193

**Published:** 2023-06-30

**Authors:** Md Firoz Anjum, Sajal Twanabasu, Sailesh Shrestha, Tashi Anjuk Lama, Dipendra Magrati

**Affiliations:** 1 Department of Paediatrics, Patan Academy of Health Sciences, Lagankhel, Lalitpur, Nepal; 2 Department of General Practice and Emergency Medicine, Patan Academy of Health Sciences, Lagankhel, Lalitpur, Nepal

**Keywords:** *basement membrane*, *case reports*, *glomerulonephritis*, *kidney*

## Abstract

Anti-glomerular basement membrane disease is an extremely uncommon entity in children. It has an incidence of 0.5 to 1 per million per year in adults and is even more uncommon in children. It occurs due to autoantibody against glomerular basement membrane collagen and is characterized by rapidly progressive glomerulonephritis with or without pulmonary hemorrhage. As the literature on anti-glomerular basement membrane disease is limited from our part of the world, it is important to consider it as the rare cause of rapidly progressive glomerulonephritis as early intervention improves prognosis. We report a case of a 10-year-old male who initially presented with glomerulonephritis and later was diagnosed with anti-glomerular basement membrane disease.

## INTRODUCTION

Anti-glomerular basement membrane (anti-GBM) disease is a rare and life-threatening autoantibody-mediated small vessel vasculitis that can affect glomerular and alveolar capillaries. It is very uncommon in children and leads to rapidly progressive glomerulonephritis (RPGN) with or without pulmonary hemorrhage.^1^ Goodpasture syndrome includes the triad of glomerulonephritis, pulmonary hemorrhage, and anti-GBM antibodies whereas Goodpasture disease is used when pulmonary hemorrhage is not present.^2^ Anti-GBM disease describes anti-glomerular basement membrane antibody with either Goodpasture's syndrome or disease. Literature related to anti-GBM in children is very limited which is usually a small number of case reports and retrospective case series.^3^ Here, we report a case of anti-GBM disease in a 10-year-old male child.

## CASE REPORT

A 10-year-old previously healthy male, who was transferred to our hospital from an outside pediatric inpatient unit presented with swelling of the face and abdominal distension for 3 days following three weeks of on-and-off fever and reddish discoloration of urine. A screening urine analysis revealed 2+ protein and plenty of red blood cells per high-power field. Further work-up also demonstrated anaemia (hemoglobin of 6.6 g/dl) and urea of 90 mg/dl and serum creatinine of 4.5 mg/dl. He was admitted to the ward at another center and the initial workup was done in the line of acute glomerulonephritis. However, serum urea and creatinine kept on increasing (Urea: 300 mg/dl and creatinine: 6.2 mg/dl) and he was transferred to Patan hospital for the need of dialysis.

On admission, examination revealed a pale child with bilateral mild pedal edema and blood pressure of 120/80 mmHg (Between 95th to 95th+12 mmHg). He was afebrile, mildly tachycardic (heart rate 115 beats/ min), and saturation of 98% in room air. There was no history of joint pain/swelling or skin rash with no history of chest pain, shortness of breath, hemoptysis, palpitation, yellowish discoloration of eyes, and altered level of consciousness.

Workup showed anaemia (hemoglobin 8.3 g/dl) with a white blood cell count (11,000/mm^3^) and platelet count (234,000/mm^3^). The serum chemistry panel was abnormal for hyperkalemia (6.4 mmol/l), metabolic acidosis (HCO_3_ of 8.8 mmol/l), calcium was 9.2 mg/ dl and other reports showed hyperphosphatemia (8.7 mg/dl), and renal failure (urea 242 mg/dl and creatinine 5.6 mg/dl). Chest x-ray showed infiltrates extending from the right hilar region to the lower zone otherwise within normal limits. Further work-up to identify the cause of glomerulonephritis showed normal complement levels (C3 125 mg/dl, C4 38 mg/ dl) normal coagulation profile, negative serology for viral etiology, and negative dsDNA and anti-neutrophil cytoplasmic antibody (ANCA). Parathyroid hormone level was elevated (125 pg/ml). The urine protein to creatinine ratio was 0.15. Urine output was 0.3 to 0.5 ml/kg/hr with a subsequent decline in further days. Renal ultrasound showed normal-sized kidneys (right kidney 9.5 cm and left kidney 9.8 cm) with increased cortical echogenicity bilaterally. The patient underwent emergent hemodialysis and amlodipine 0.15 mg/kg/ day for hypertension.

**Figure 1:**
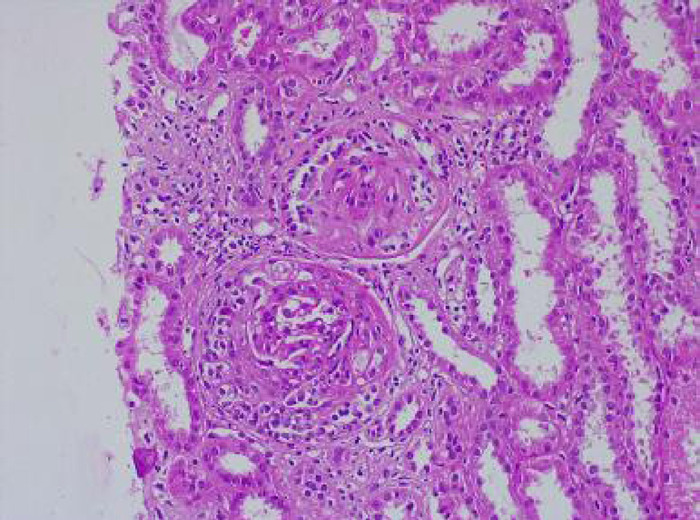
Renal biopsy showing crescents.

A renal biopsy was done. 16 glomeruli were available for light microscopic examination and none of the glomeruli showed global sclerosis. All glomeruli revealed overlying partial/circumferential cellular/fibro cellular crescents (Figure 1).

Six out of 16 glomeruli showed segmental fibrinoid tuft necrosis. Mild chronic interstitial inflammation was observed with acute tubular injury. Immunofluorescence showed intense linear glomerular capillary staining with IgG, kappa, and lambda chains. The renal biopsy findings were suspicious of anti-GBM mediated crescentic glomerulonephritis. The patient was started on high-dose methylprednisolone 30 mg/kg/day. Anti-GBM titers (IgG antibody) were raised 69 RU/ml (51-256 RU/ml: strongly positive). Unfortunately, the facility of plasmapheresis was not available at the Patan hospital, and renal function was not improving despite alternate-day hemodialysis. He received the first dose of cyclophosphamide for further immunosuppression. The patient was counseled and referred to a center with a plasmapheresis facility.

## DISCUSSION

Anti-GBM disease is a very rare condition with an incidence of 0.5 to 1 per million per year in adults and even more uncommon in children.^4^ However, it is responsible for around 20% of all causes of RPGN and 3% of crescentic glomerulonephritis in children.^5^

RPGN is the classical presentation of anti-GBM disease with 80-90% requiring acute kidney replacement therapy.^6^ However, many patients report nonspecific signs and symptoms like fever, myalgia, and arthralgia before the classical presentation. Pulmonary hemorrhage might be present in 60% of the cases with very few cases presenting with pulmonary manifestations alone.^7^ Renal manifestations can range from hematuria and proteinuria to rapidly progressing renal failure with oliguria, fluid overload, and severe hypertension. In our case, the initial presentation was hematuria which rapidly progressed to renal failure. There was no pulmonary involvement in the case reported. Exposure to smoke appears to be a strong factor in the development of anti-GBM disease though; no such relationship has been demonstrated in pediatric cases.

The diagnosis of anti-glomerular basement membrane disease is made when anti-GBM antibodies are detected either in circulation or in the tissue using renal or pulmonary biopsies. A renal biopsy can be used to confirm the diagnosis. Light microscopy usually shows crescentic glomerulonephritis but the characteristic linear IgG deposition along the capillary wall in immunofluorescence microscopy is diagnostic. Anti-GBM titers (IgG antibody) were raised 69 RU/ml (51-256 RU/ml: strongly positive) in our case and renal biopsy findings were also consistent with the anti-GBM disease. There are many case reports with dual ANCA and anti-GBM positivity in children, however, ANCA was negative in our case.^8^

Plasmapheresis, which removes circulating antibodies and potential immune mediators is the initial treatment of choice. Corticosteroids and cyclophosphamide are the preferred immunosuppressive therapy. High-dose methylprednisolone and cyclophosphamide were used in our case. Unfortunately, as the plasmapheresis facility is not available at Patan Hospital; the child was referred. The facility of plasmapheresis is very limited even in Kathmandu valley. Rituximab can be tried as an alternative immunosuppressant. Supportive therapy includes the use of renal replacement therapy; usually, hemodialysis, and anti-hypertensive agents when required. The child in our case was on hemodialysis and amlodipine was started for hypertension.

Anti-GBM antibodies should be monitored weekly until two negatives are achieved then after monthly for 6 months. As plasmapheresis could not be initiated in our patient, we did not repeat the anti-GBM antibody. Repeat anti-GBM is planned during the follow-up of the patient. Once remission is achieved, low-dose prednisolone, azathioprine, or mycophenolate may be used for maintenance.^9^

The mortality rate is 20% in adults and 30% in children. The prognosis for renal recovery is worse in the presence of oliguria, presenting creatinine >6.8 or renal biopsy showing >50% crescent formation within glomeruli at the time of diagnosis.^1,2^ Prognosis of renal recovery in the patient reported is worse as the child presented with oliguria with borderline serum creatinine (5.6 mg/dl) and renal biopsy showing overlying partial/circumferential cellular/fibro cellular crescents in 16 out of 16 glomeruli.

Anti-GBM is a rare clinical entity in a pediatric population. It should be considered in a differential diagnosis in any case of glomerulonephritis with rapid progression to renal failure with or without pulmonary manifestation. As the literature on anti-GBM is limited from our part of the world, it is important to consider anti-GBM as the rare cause of RPGN as early intervention improves prognosis.
